# Photothermal treatment of port-wine stains using erythrocyte-derived particles doped with indocyanine green: a theoretical study

**DOI:** 10.1117/1.JBO.23.12.121616

**Published:** 2018-11-29

**Authors:** Joshua M. Burns, Wangcun Jia, J. Stuart Nelson, Boris Majaron, Bahman Anvari

**Affiliations:** a University of California, Riverside, Department of Bioengineering, Riverside, California, United States; b University of California, Irvine, Beckman Laser Institute and Medical Clinic, Irvine, California, United States; c Jožef Stefan Institute, Department of Complex Matter, Ljubljana, Slovenia; d University of Ljubljana, Faculty of Mathematics and Physics, Ljubljana, Slovenia

**Keywords:** laser therapy, mathematical modeling, near-infrared, optical materials, red blood cells, skin

## Abstract

Pulsed dye laser irradiation in the wavelength range of 585 to 600 nm is currently the gold standard for treatment of port-wine stains (PWSs). However, this treatment method is often ineffective for deeply seated blood vessels and in individuals with moderate to heavy pigmentation. Use of optical particles doped with the FDA-approved near-infrared (NIR) absorber, indocyanine green (ICG), can potentially provide an effective method to overcome these limitations. Herein, we theoretically investigate the effectiveness of particles derived from erythrocytes, which contain ICG, in mediating photothermal destruction of PWS blood vessels. We refer to these particles as NIR erythrocyte-derived transducers (NETs). Our theoretical model consists of a Monte Carlo algorithm to estimate the volumetric energy deposition, a finite elements approach to solve the heat diffusion equation, and a damage integral based on an Arrhenius relationship to quantify tissue damage. The model geometries include simulated PWS blood vessels as well as actual human PWS blood vessels plexus obtained by the optical coherence tomography. Our simulation results indicate that blood vessels containing micron- or nano-sized NETs and irradiated at 755 nm have higher levels of photothermal damage as compared to blood vessels without NETs irradiated at 585 nm. Blood vessels containing micron-sized NETs also showed higher photothermal damage than blood vessels containing nano-sized NETs. The theoretical model presented can be used in guiding the fabrication of NETs with patient-specific optical properties to allow for personalized treatment based on the depth and size of blood vessels as well as the pigmentation of the individual’s skin.

## 1 Introduction

Port-wine stains (PWSs) are congenital and progressive malformations of dermal capillaries that occur in approximately three children per 1000 live births.^1^ Pulsed dye lasers (PDL) with wavelengths in the range of 585 to 600 nm have become the most prevalent treatment for PWSs.^2^^–^^5^ This treatment uses laser light to target the endogenous hemoglobin for photothermolysis of the blood vessels (BVs). Although clinical results can be obtained in the treatment of light red or red macular PWS, especially in children and light-skin individuals, clearing is inefficient, requiring multiple therapeutic sessions. It is reported that about 60% of patients receive reduction in redness and lesion size after 10 treatment sessions, but only 10% to 20% experience complete clearance of the stain.^4^^,^^6^ In addition, PDL treatments within safe therapeutic radiant dosages of ≈7 to $16\;J/{\mathrm{cm}}^{2}$, when used in combination with cryogen spray cooling of skin,^7^ do not provide the clinically desirable photothermal effects to completely destroy BVs located at depths greater than about 500  μm below skin surface.

Another challenge with the current approach is related to the treatment of patients with moderate to heavy skin pigmentation (Fitzpatrick types IV to VI). The skins of these patients contain a large number of melanosome organelles within the melanocytes, located primarily in the basal epidermis layer. Melanosomes contain melanin, a polymeric pigment with broad absorption spectrum ranging from UV to visible, including the 585- to 600-nm treatment band. Therefore, melanin acts as a chromophore competing with hemoglobin to partially absorb the photons intended to reach the dermal vascular plexus. The result is nonspecific thermal injury to the epidermis and inefficient photodestruction of target BVs.

A potential phototherapeutic approach is to use an exogenous absorber that can be activated by near-infrared (NIR) light to generate heat. The advantage of changing the therapeutic wavelength from 585 nm to NIR wavelength of 755 nm is associated with nearly threefold reduction in the absorption coefficient of a single melanosome.^8^ One particularly promising exogenous absorber is indocyanine green (ICG). It is one of the least toxic agents administered to humans and the only FDA-approved NIR dye for specific imaging applications.^9^^,^^10^ ICG has also been investigated as a photothermal agent for treatment of PWSs^11^^,^^12^ and as a photosensitizer for photodynamic therapy of choroidal melanomas and breast adenocarcinomas.^13^^,^^14^

Despite its current use in clinical settings, ICG’s major disadvantage is its short half-life in blood (3 to 4 min).^9^ Due to its amphiphilic nature, ICG can nonspecifically bind to various biomolecules in the blood, including high- and low-density lipoproteins and albumin. Hepatic parenchymal cells uptake ICG and/or the ICG-bound molecular complexes leading to biliary excretion into the intestine.^15^

To extend the circulation time and reduce nonspecific interactions with plasma proteins and other biomolecules, ICG has been encapsulated into various constructs including synthetic polymers,^16^^–^^19^ micelles,^20^^–^^22^ liposomes,^23^^–^^25^ and silica/silicate matrices.^26^^,^^27^ Encapsulation within constructs derived from erythrocytes are particularly advantageous due to their potential biocompatibility.

We have previously reported on fabrication of vesicles derived from erythrocytes and loaded with ICG, and their utility for fluorescence imaging and photodestruction of human cells.^28^ We refer to these constructs as NIR erythrocyte-mimicking transducers (NETs). A particular feature of NETs is that their diameter can be tuned from micron- to nano-scale, and their ICG content can be adjusted independently during the fabrication process. We recently characterized the effects of NETs’ diameter and ICG concentration on the resulting optical properties, absorption coefficient ($\mu_{a}$), and reduced scattering coefficient ($\mu_{s}^{\prime }$) of these constructs.^29^ These results are particularly useful in development of mathematical models aimed at quantification of light and laser-induced temperature distributions in tissues containing NETs. As a first step toward potential application of NETs in phototherapy of PWSs, we analyze in dedicated numerical simulations the photothermal response of BVs containing NETs, and compare the results with those obtained with the current treatment approach of using PDL in 585- to 600-nm range. These results can be used to identify the optimum formulation of NETs and appropriate irradiation parameters for NIR photothermal treatment of PWSs.

## 2 Materials and Methods

### 2.1 Overview of Model

**Fig. 1:**
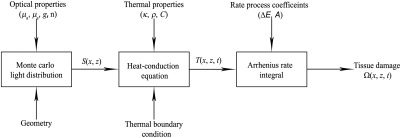
Block diagram of the mathematical model components.

In Fig. 1, we present a block diagram of the mathematical model consisting of three components: a Monte Carlo light-transport model to estimate the energy deposition in response to laser irradiation,^30^^,^^31^ finite-element model to compute the spatiotemporal temperature distribution based on the heat diffusion equation, and an Arrhenius rate process integral to quantify the resulting thermal damage. Each component of the model is described in the following sections.

### 2.2 Model Geometry

**Fig. 2:**
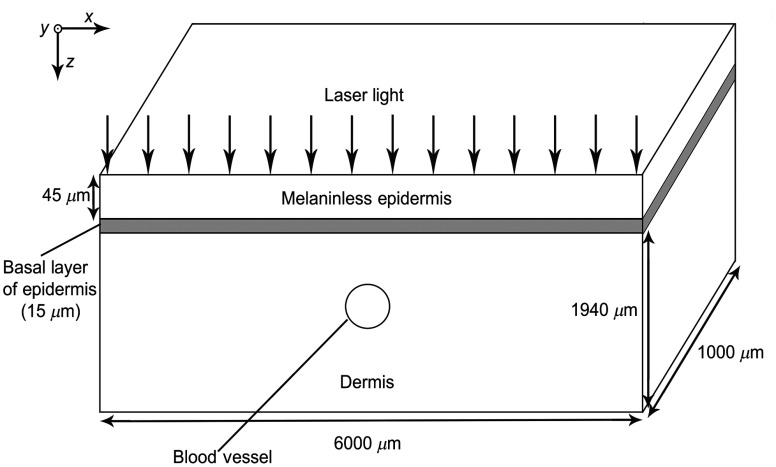
Simulated PWS geometry.

We used two geometries to model the human skin with PWS. The first geometry simulated the skin and consisted of a 60-μm-thick epidermis and 1940-μm-thick dermis containing BVs (Fig. 2). The epidermis was composed of a 45-μm melaninless layer and a 15-μm melanocyte-filled layer located at the basal epidermis. BVs were assumed to be cylinders with diameters of 200  μm, running parallel to the y-axis for the entire length of the geometry (1 mm). The top position of the vessel was located either at 500 or 800  μm below skin surface.

**Fig. 3:**
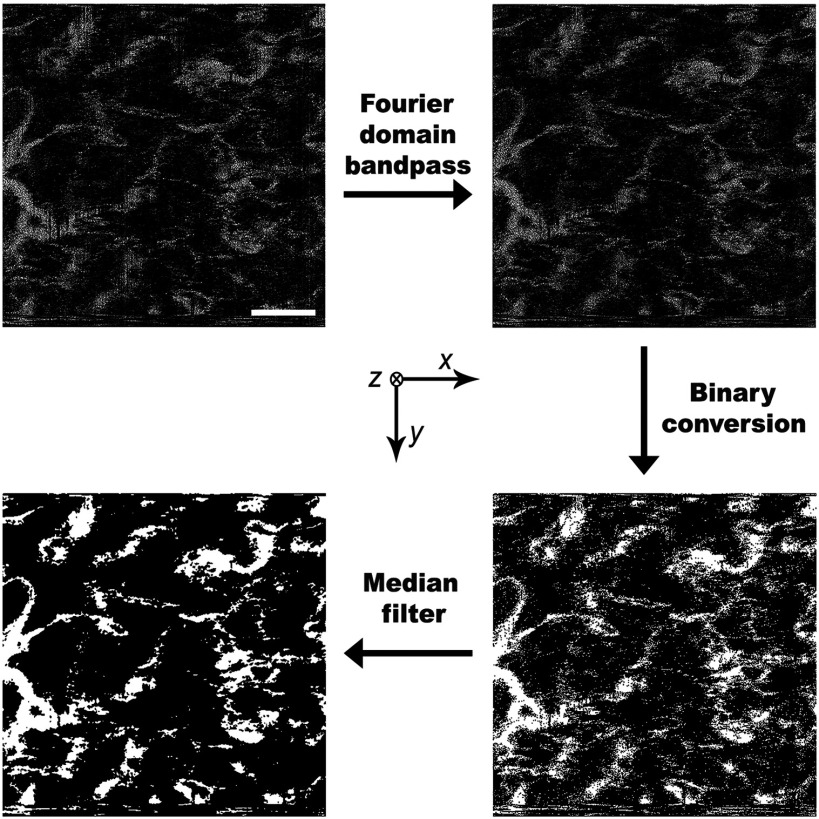
Flowchart of processing an OCT image of PWS skin for use in mathematical model. With respect to the coordinate system shown in Fig. 2, the coordinate system here is rotated 90 deg counterclockwise around the x-axis. The images are horizontal cross-sections in the x-y plane at depth of z=680  μm.

In addition, we also constructed a PWS skin geometry from an image obtained by optical coherence tomography (OCT) of a PWS patient (Fig. 3).^32^ We first removed motion artifact associated with the image cube, 5 mm (length, in x-direction) ×5  mm (width, in y-direction) ×2.8  mm (height, in z-direction), by taking the Fourier transform of each two-dimensional (2-D) section (5  mm×5  mm) (axial resolution = 8  μm) and applying a bandpass filter with cutoff normalized frequencies of 0.1 and 1.0, where 1.0 corresponds to half of the sampling frequency in the horizontal direction, and 0.0 and 0.01 in the vertical direction. We then took the inverse Fourier transform of each image and converted into binary values through thresholding. Finally, speckle noise was removed using a three-dimensional (3-D) median filter.

### 2.3 Optical Properties of Human Skin

The baseline absorption coefficient for melaninless epidermis $\mu_{a,\text{base}}$ (${\mathrm{mm}}^{-1}$) and bloodless dermis was approximated by^33^

$$\mu_{a,\text{base}}=0.0244+8.53\cdot \exp(-\frac{\lambda -154}{66.2}),$$ (1)

where λ is the wavelength in nm. The absorption coefficient for the basal layer of the epidermis $\mu_{a,\mathrm{basal}}$ is a combination of the $\mu_{a,\mathrm{base}}$ and the absorption coefficient of melanosomes $\mu_{a,\mathrm{mel}}$:

$$\mu_{a,\mathrm{basal}}=f_{\text{mel}}\cdot \mu_{a,\mathrm{mel}}+(1-f_{\text{mel}})\cdot \mu_{a,\text{base}},$$ (2)

where $f_{\text{mel}}$ is the volume fraction of melanosomes. In this study, we present lightly, moderately, and heavily pigmented skin with $f_{\text{mel}}=4\%$, 15%, and 50%, respectively,^34^ and $\mu_{a,\mathrm{mel}}({\mathrm{mm}}^{-1})$ defined as^33^

$$\mu_{a,\mathrm{mel}}=6.6\times 10^{10}\cdot \lambda^{-3.33}.$$ (3)

For simulated single vessels, we accounted for attenuation of the incident light by absorption in the remaining PWS BVs by adjusting the absorption coefficient of the dermis ($\mu_{a,\mathrm{der}}$) (Table 1) as^30^

$$\mu_{a,\mathrm{der}}=f_{i}C_{i}\mu_{a,\mathrm{blood}}+(1-f_{i})\mu_{a,\mathrm{der}},$$ (4)

where $C_{i}$ is the correction factor accounting for optical screening within a BV with radius $r_{i}$, and $f_{i}$ is the fractional volume occupied by the PWS BVs. In this adjustment, we account for the average $r_{i}$ of the PWS BVs within a range of subsurface depths (z) (see Table 1) and assume 45% hematocrit (hct) with oxygen saturation of 70%:^30^

$$C_{i}\approx 0.039+0.486\exp[-\frac{\mu_{a,\mathrm{blood}}(45\%\mathrm{hct})r_{i}}{0.193}]+0.468\exp[-\frac{\mu_{a,\mathrm{blood}}(45\%\mathrm{hct})r_{i}}{0.914}].$$ (5)

**Table 1:** Adjusted absorption coefficient of dermis used in this study.

z (μm)	$r_{i}$ (μm)	$f_{i}$(%)	$\mu_{a,\mathrm{der}}$ (585 nm) (${\mathrm{mm}}^{-1}$)	$\mu_{a,\mathrm{der}}$ (755 nm) (${\mathrm{mm}}^{-1}$)
60 to 260	25	5.2	0.071	0.025
260 to 460	37	8.0	0.090	0.024
460 to 660	34	3.8	0.062	0.025
660 to 860	27	2.5	0.053	0.025
860 to 1060	24	2.0	0.050	0.025
1060 to 2000	24	1.5	0.047	0.025
PWS blood vessels imaged by OCT all z (μm)			0.073	0.026

The adjusted values of $\mu_{a,\mathrm{der}}$ at 585 nm vary with depth and the corresponding values of $r_{i}$ and $f_{i}$, whereas those for 755 nm are nearly independent of depth.

For OCT simulated vessels, $\mu_{a,\mathrm{der}}$ was expressed as

$$\mu_{a,\mathrm{der}}=f_{\text{blood}}\cdot \mu_{a,\mathrm{blood}}+(1-f_{\text{blood}})\cdot \mu_{a,\mathrm{bas}},$$ (6)

where $f_{\text{blood}}$ is the volume fraction of blood in the healthy dermis, chosen to be 0.2% consistent with literature reported value,^33^ and $\mu_{a,\mathrm{blood}}$ (${\mathrm{mm}}^{-1}$) is the absorption coefficient of blood with oxygen saturation of 70%. In this case, values of $\mu_{a,\mathrm{der}}$ were not adjusted since the actual distributions of BVs were directly obtained from the OCT images.

The scattering coefficients ($\mu_{s}$) (${\mathrm{mm}}^{-1}$) of the melaninless epidermis, epidermal basal layer, and dermis were estimated as^33^

$$\mu_{s}=\frac{2\times 10^{4}\cdot \lambda^{-1.5}+2\times 10^{11}\cdot \lambda^{-4}}{1-g},$$ (7)

where the scattering anisotropy factor (g) was assumed to be 0.8 for 585 nm^35^ and 0.91 at 755 nm,^36^ the irradiation wavelengths used in this study.

### 2.4 Optical Properties of NETs and Blood Vessels

Spectrally dependent values of $\mu_{a}$ and $\mu_{s}^{\prime }$ for micron-sized NETs (μNETS) and nano-sized NETs (nNETs) with respective mean diameters ($d_{\text{mean}}$) of ≈4  μm and 92 nm were previously estimated using an integrating sphere in conjunction with an inverse adding-doubling algorithm.^29^ Values of $\mu_{a}$ and $\mu_{s}$ for blood with 45% hct and oxygen saturation of 70% were obtained from the literature.^37^ We then determined the effective optical properties of BV as

$$\mu_{a,\mathrm{BV}}=(1-f_{\mathrm{NETs}})\mu_{a,\mathrm{blood}}+f_{\mathrm{NETs}}\mu_{a,\mathrm{NETs}},$$ (8)

$$\mu_{s,\mathrm{BV}}=(1-f_{\mathrm{NETs}})\mu_{s,\mathrm{blood}}+f_{\mathrm{NETs}}\mu_{s,\mathrm{NETs}},$$ (9)

$$g_{\mathrm{BV}}=(1-f_{\mathrm{NETs}})g_{\mathrm{blood}}+f_{\mathrm{NETs}}g_{\mathrm{NETs}},$$ (10)

where $\mu_{a,x},\mu_{s,x}$, and $g_{x}$ are the respective effective absorption and scattering coefficients, and anisotropy factor, with the subscript x standing for either BVs containing blood and volume fraction of NETs ($f_{\text{NETs}}$) (assumed 10% or 25%), blood, or NETs. We varied $\mu_{a,\mathrm{NETs}}$ so that $\mu_{a,\mathrm{BV}}$ had values in the range of 1 to $18\;{\mathrm{mm}}^{-1}$. To determine the ICG concentration in NETs fabrication buffer [ICG] (μM) to produce a desired $\mu_{a,\mathrm{BV}}$, we first used a linear regression to quantify the relationship between the previously reported estimates of $\mu_{a,\mathrm{NETs}}$, and the utilized values of [ICG]

$$[\mathrm{ICG}]=17.8\cdot \mu_{a,\mathrm{NETs}}.$$ (11)

Next, by rearranging Eq. (8) for $\mu_{a,\mathrm{NETs}}$, and substituting the expression into Eq. (11), we obtained [ICG]:

$$[\mathrm{ICG}]=17.8[\frac{\mu_{a,\mathrm{BV}}-(1-f_{\mathrm{NETs}})\mu_{a,\mathrm{blood}}(45\%\mathrm{hct})}{f_{\mathrm{NETs}}}].$$ (12)

Using Eq. (12), we determined [ICG] values of ≈108, 286, 464, 642, 820, and 998  μM to produce the corresponding $\mu_{a,\mathrm{BV}}$ values of 1, 2, 3, 4, 5, and $6\;{\mathrm{mm}}^{-1}$. For example, values of $f_{\text{NETs}}=25\%$ and $\mu_{a,\mathrm{BV}}=18\;{\mathrm{mm}}^{-1}$ resulted in [ICG] = 1258  μM. Values of the optical properties used in this study are provided in Table 2.

**Table 2:** Optical properties used in this study. Optical properties for blood are based on 45% hematocrit.

Wavelength	Optical properties	Melaninless epidermis	Basal layer containing melanosomes (light)	Basal layer containing melanosomes (moderate)	Basal layer containing melanosomes (heavy)	Dermis	Blood	μNETs ($d_{\text{mean}}=4\;\mu m$)	nNETs ($d_{\text{mean}}=92\;\mathrm{nm}$)	$\mu_{a,\mathrm{BV}}$
585 nm	$\mu_{a}$ (${\mathrm{mm}}^{-1}$)	0.037	1.65	6.07	20.15	a	17.91	—	—	
	$\mu_{s}$ (${\mathrm{mm}}^{-1}$)	15.61	15.61	15.61	15.61	15.61	76.05	—	—	
	g	0.80	0.80	0.80	0.80	0.80	0.970	—	—	
755 nm	$\mu_{a}$ (${\mathrm{mm}}^{-1}$)	0.025	0.71	2.58	8.61	a	0.44	6.04 to 56.04	6.04 to 56.04	1 to 18
	$\mu_{s}$ (${\mathrm{mm}}^{-1}$)	17.55	17.55	17.55	17.55	17.55	79.85	2.20	0.06	59.90 to 72.09
	g	0.91	0.91	0.91	0.91	0.91	0.983	0.99	0.55	0.87 to 0.98

a See Table 1.

### 2.5 Mathematical Model

The 3-D Monte Carlo model for light transport and energy deposition was developed earlier by Majaron et al.^30^^,^^31^ Our simulations involved one million photons for 585- or 755-nm flat-top laser beams with diameter of 6 mm for simulated PWS geometry, and 8 mm for the skin model based on the PWS patient OCT record. With a 2.3-GHz central processing unit, each Monte Carlo simulation took ≈2 to 11 h depending on the geometry and optical properties of the tissue. For example, the run time was ≈10.5  h for a simulated geometry containing nNETs ($f_{\text{NETs}}=10\%$, $\mu_{a,\mathrm{BV}}=6\;{\mathrm{mm}}^{-1}$), and lightly pigmented skin ($f_{\text{mel}}=4\%$) irradiated at 755 nm. For an OCT geometry, an example run time was ≈2.0  h for a moderately pigmented skin ($f_{\text{mel}}=15\%$) irradiated at 585 nm without NETs.

We used the heat diffusion equation to calculate the spatiotemporal temperature profiles in response to laser irradiation:

$$\kappa [\frac{\partial T^{2}(x,z,t)}{\partial^{2}x}+\frac{\partial T^{2}(x,z,t)}{\partial^{2}z}]+S(x,z)=\rho C\frac{\partial T(x,z,t)}{\partial t},$$ (13)

where κ is the thermal conductivity, T(x,z,t) is the skin temperature (°C) (assuming symmetry about the y-axis), x is the lateral distance from the beam center (m), z is the depth into the skin, t is time (s), S(x,z) is the heat source term resulting from absorption of photons ($W\;m^{-3}$), ρ is the density ($\mathrm{kg}\;m^{-3}$), and C is the specific heat capacity ($J\;{\mathrm{kg}}^{-1}{}^{\circ}C^{-1}$). Thermal properties of human skin used in the study were density, $\rho =1200\;\mathrm{kg}\;m^{-3}$, specific heat capacity, $C=3600\;J\;{\mathrm{kg}}^{-1}{}^{\circ}C^{-1}$, and κ=0.26, 0.53, $0.53\;W\;m^{-1}{}^{\circ}C^{-1}$ for epidermis, dermis, and BVs, respectively.^38^ The baseline skin temperature was 35°C.

We assumed that a cryogen spurt was sprayed onto the skin surface immediately prior to the laser pulse.^39^^–^^43^ To account for this type of skin cooling, we implemented a convective surface boundary condition as follows:

$$-\kappa \frac{\partial T(x,z,t)}{\partial z}{|}_{z=0}=h[T_{\mathrm{med}}-T(x,z,t){|}_{z=0}],$$ (14)

where $T_{\text{med}}$ is the temperature of the cryogen film on the skin surface (°C), and h is the convective heat transfer coefficient at the skin surface. We utilized the relevant thermal parameters associated with the boundary condition from our previous work^43^^–^^45^ and list them in Table 3. The heat-diffusion equation was solved numerically using the commercial finite-elements software package, FEMLAB^®^. The finite-elements run time for simulated geometries was 2 to 3 min using 1200×400 nodes. For OCT geometries, run time was 12 to 18 h using 500×350 nodes.

**Table 3:** Parameters for surface thermal boundary condition.

Time interval	h ($W/m^{2}{}^{\circ}C$)	$T_{\text{surface}}$ (°C)	Duration (ms)
Cryogen spurt application	4000	−50	100
Cryogen pool residence	3000	−26	200
Rewarming	10	25	500

Thermal damage to the skin was quantified using the Arrhenius rate damage integral

$$\Omega (x,z)=A\int_{0}^{\tau }\exp[-\frac{E}{RT(x,z,t)}]dt,$$ (15)

where Ω(x,z) is the damage index, A is the frequency factor ($A=1.8\times 10^{51}\;s^{-1}$ for bulk skin and $A=7.6\times 10^{66}\;s^{-1}$ for blood), E is the damage activation energy ($E=327,000\;J\;{\mathrm{mol}}^{-1}$ for bulk skin and $E=455,000\;J\;{\mathrm{mol}}^{-1}$ for blood), and R is the universal gas constant ($R=8.314\;J\;{\mathrm{mol}}^{-1}\;K^{-1}$).^46^^,^^47^ Value of Ω(x,z)=1 is associated with irreversible damage to 63% of the tissue. Laser pulse duration was fixed at 3 ms, and it was applied immediately after termination of a 100-ms cryogen spurt. Values of Ω(x,z) were calculated following the laser pulse and through the duration of the rewarming interval. The percent photothermal damage to the BV (% damage) was defined as the area of the damaged vessel at this time divided by the total cross-sectional area.

## 3 Results and Discussion

### 3.1 Simulated PWS Geometry

#### 3.1.1 Threshold incident dosage for epidermal damage in absence of NETs

We first determined the threshold incident dosage ($D_{\mathrm{th}}$) for epidermal damage in response to 585 and 755 nm irradiation without BVs. For lightly, moderately, and heavily pigmented skin, values of $D_{\mathrm{th}}$ were determined as 8, 3, and $1\;J/{\mathrm{cm}}^{2}$ for 585 nm, and 21, 6, and $3\;J/{\mathrm{cm}}^{2}$ for 755-nm irradiation, respectively (Table 4). These simulation results confirm that use of 755 nm increases the threshold for epidermal injury, due to the lower absorption of melanin at this wavelength compared to 585 nm. These results are consistent with a previous clinical study where laser irradiation at 755 nm with incident dosages greater than $10\;J/{\mathrm{cm}}^{2}$ effective induced PWS lightening.^48^

**Table 4:** Estimated threshold incident dosages for epidermal damage ($D_{\mathrm{th}}$) in absence of NETs for simulated PWS skin geometry.

Wavelength	Skin pigmentation	$f_{\mathrm{mel}}$ (%)	$D_{\mathrm{th}}$ ($J\;{\mathrm{cm}}^{-2}$)
585 nm	Light	4	8
	Moderate	15	3
	Heavily	50	1
755 nm	Light	4	21
	Moderate	15	6
	Heavily	50	3

#### 3.1.2 Photothermal response of PWS blood vessels in the absence of NETs

**Fig. 4:**
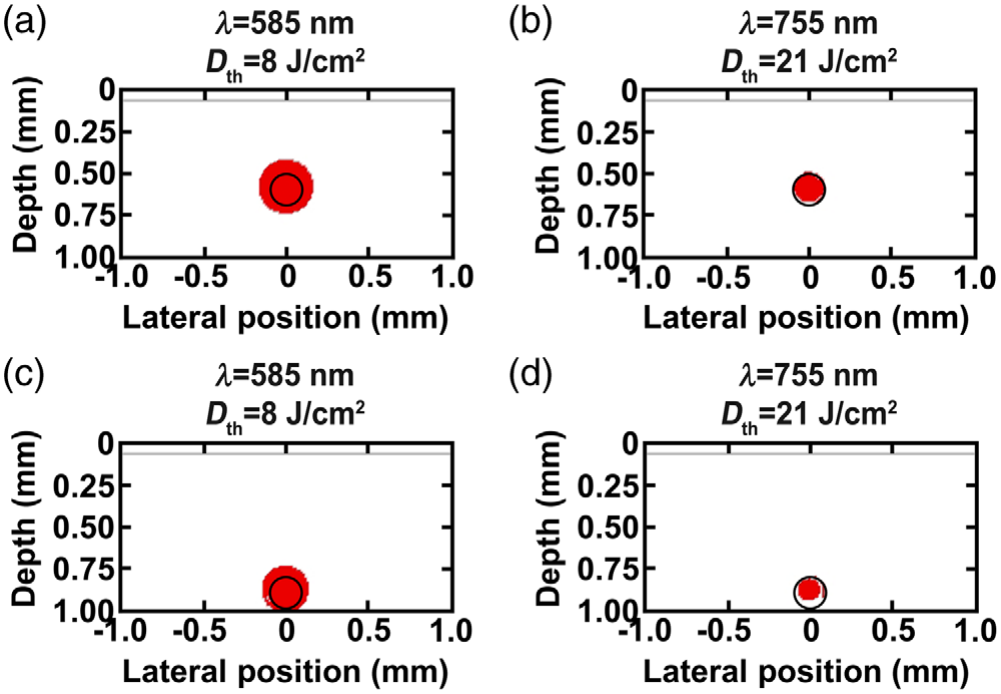
Damage profiles shown in red (Ω≥1) for blood vessels located at (a, b) 500 and (c, d) 800  μm below skin surface for lightly pigmented skin ($f_{\text{mel}}=4\%$) in response to (a, c) 585 and (b, d) 755-nm laser irradiation without NETs.

The percent photothermal damage profiles to BVs without NETs for lightly ($f_{\text{mel}}=4\%$) pigmented skin at the $D_{\mathrm{th}}=8\;J/{\mathrm{cm}}^{2}$ at 585 nm and $21\;J/{\mathrm{cm}}^{2}$ at 755 nm are shown in Fig. 4. Complete damage to BVs without NETs was observed for BVs in response to 585-nm laser irradiation at $D_{\mathrm{th}}=8\;J/{\mathrm{cm}}^{2}$ [Figs. 4(a) and 4(c)]. However, this vascular damage was also accompanied by damage to the surrounding dermis. For BVs without NETs irradiated at 755 nm, there was 79 and 50% damage to vessels at depths of 500 and 800  μm, respectively, without thermal injury to perivascular dermis [Figs. 4(b) and 4(d)].

**Fig. 5:**
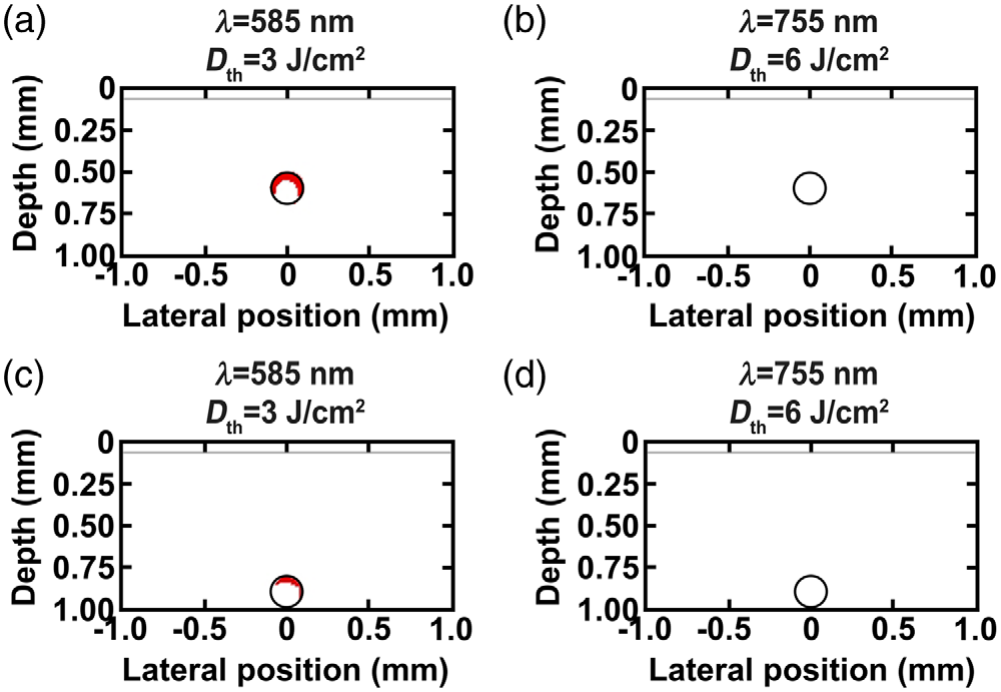
Damage profiles shown in red (Ω≥1) for blood vessels located at (a, b) 500 and (c, d) 800  μm below skin surface for moderately pigmented skin ($f_{\text{mel}}=15\%$) in response to (a, c) 585 and (b, d) 755-nm laser irradiation without NETs.

For moderately pigmented skin ($f_{\text{mel}}=15\%$) in response to 585-nm irradiation at $D_{\mathrm{th}}=3\;J/{\mathrm{cm}}^{2}$, % damage to the vessels at 500 and 800  μm were 34% and 11%, respectively (Fig. 5). In response to 755-nm irradiation at $D_{\mathrm{th}}=6\;J/{\mathrm{cm}}^{2}$, there was no damage to the BVs at either depth. Lastly, for heavily pigmented skin ($f_{\text{mel}}=50\%$) in response to 585-nm irradiation at $D_{\mathrm{th}}=1\;J/{\mathrm{cm}}^{2}$ and 755 nm irradiation at $D_{\mathrm{th}}=3\;{\mathrm{cm}}^{2}$, there was no damage to the vessels at either depth (results not shown). The decrease in % damage for blood vessels with moderately and heavily pigmented skin can be attributed to the decrease in $D_{\mathrm{th}}$, as well as increased light absorption within the basal epidermis.

#### 3.1.3 Photothermal response of PWS blood vessels containing NETs

**Fig. 6:**
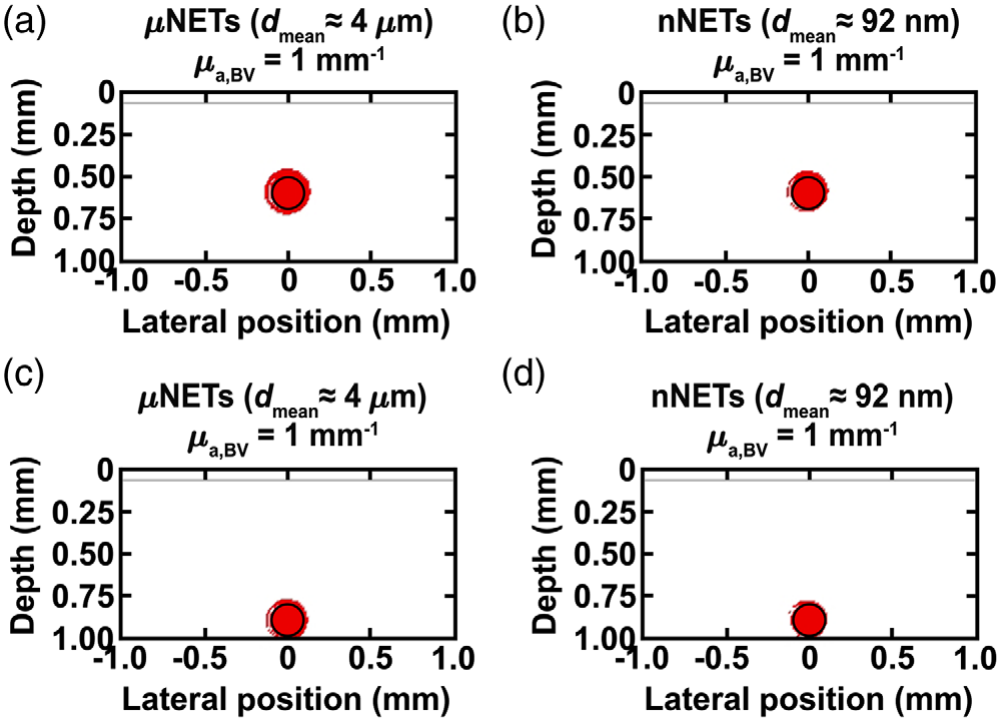
Damage profiles shown in red (Ω≥1) for blood vessels containing μNETs, or nNETs at $f_{\text{NETs}}=10\%$ with $\mu_{a,\mathrm{BV}}=1\;{\mathrm{mm}}^{-1}$, and located at depths of (a, b) 500 and (c, d) 800  μm below skin surface. Light skin pigmentation ($f_{\text{mel}}=4\%$), λ=755  nm, and $D_{\mathrm{th}}=21\;J/{\mathrm{cm}}^{2}$.

The percent photothermal damage profiles to blood vessels containing μNETs or nNETs ($f_{\mathrm{NETs}}=10\%$) for lightly pigmented skin ($f_{\text{mel}}=4\%$) in response to 755 nm irradiation at $D=21\;J/{\mathrm{cm}}^{2}$ are shown in Fig. 6. There was 100% damage to blood vessels located at depths of 500 and 800  μm below skin surface containing μNETs, or nNETs with $\mu_{a,\mathrm{BV}}$ of $1\;{\mathrm{mm}}^{-1}$ (Fig. 6). As the depth of the blood vessel increased from 500 to 800  μm, there was a decrease in damage to the dermis, which was further reduced when using nNETs as compared to μNETs. These results suggest PWS patients with light pigmentation and blood vessels as deep as 500 to 800  μm could potentially benefit from the delivery of μNETs or nNETs that yield $\mu_{a,\mathrm{BV}}=1\;{\mathrm{mm}}^{-1}$ to achieve photothermal injury to the blood vessels. This value of $\mu_{a,\mathrm{BV}}$ corresponds to μNETs or nNETs with $\mu_{a,\mathrm{NETs}}\approx 6\;{\mathrm{mm}}^{-1}$, which can be fabricated by using ≈108  μM ICG in the fabrication buffer [Eq. (12)].

**Fig. 7:**
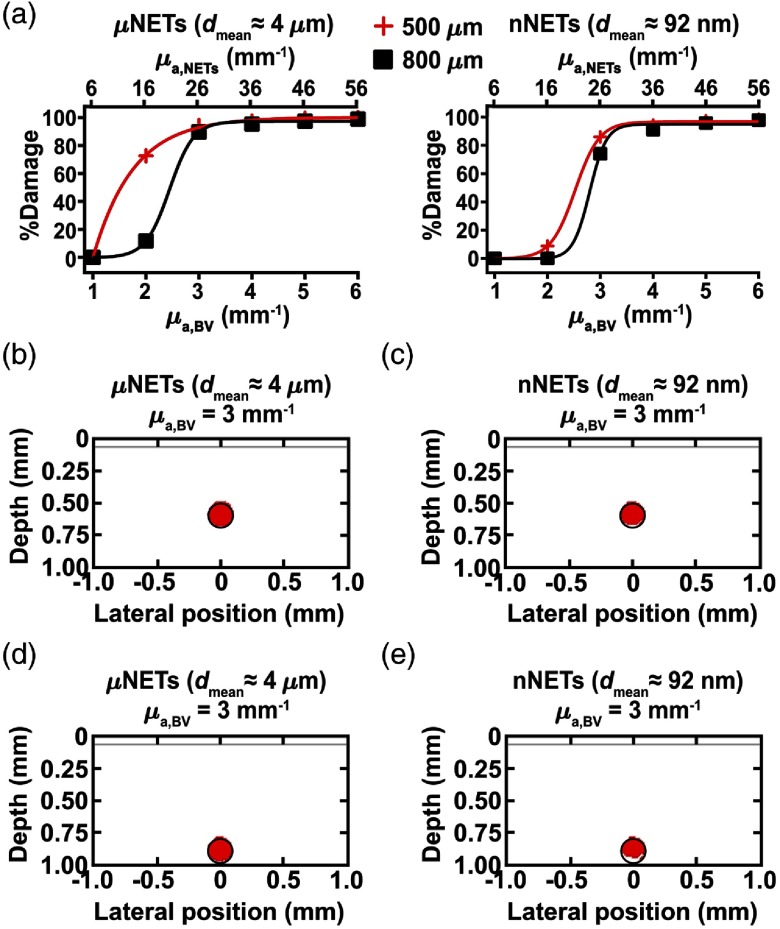
(a) % Damage to blood vessels containing μNETs or nNETs ($f_{\text{NETs}}=10\%$) as a function of $\mu_{a,\mathrm{BV}}$ and $\mu_{a,\mathrm{NETs}}$ (markers) fitted with exponential or sigmoidal functions (solid curves). Damage profiles shown in red (Ω≥1) for blood vessels located at depths of (b, c) 500 and (d, e) 800  μm below skin surface containing μNETs, or nNETs at $f_{\text{NETs}}=10\%$ with $\mu_{a,\mathrm{BV}}=3\;{\mathrm{mm}}^{-1}$. Moderate skin pigmentation ($f_{\text{mel}}=15\%$), λ=755  nm, and $D_{\mathrm{th}}=6\;J/{\mathrm{cm}}^{2}$.

For moderately pigmented skin ($f_{\text{mel}}=15\%$), $f_{\text{NETs}}=10\%$, and 755-nm laser irradiation at $D_{\mathrm{th}}=6\;J/{\mathrm{cm}}^{2}$, there was no damage to the blood vessels when using μNETs or nNETs that yielded $\mu_{a,\mathrm{BV}}=1\;{\mathrm{mm}}^{-1}$ [Fig. 7(a)]. For $\mu_{a,\mathrm{BV}}=3\;{\mathrm{mm}}^{-1}$, the blood vessel at depth of 500  μm showed 94 and 86% damage when containing μNETs or nNETs, respectively [Figs. 7(a) and 7(b)]. For this value of $\mu_{a,\mathrm{BV}}$, when the depth of the blood vessel was increased to 800  μm, respective % damage values were reduced to 89% and 74% in the presence of μNETs or nNETs [Figs. 7(c) and 7(d)].

In general, for the case of the blood vessel containing μNETs at depth of 500  μm, % damage to blood vessels followed an exponentially increasing behavior with $\mu_{a,\mathrm{BV}}$, and approached an asymptotic value of ≈95% for values of $\mu_{a,\mathrm{BV}}\geq 3\;{\mathrm{mm}}^{-1}$, corresponding to $\mu_{a,\mathrm{NETs}}\geq 26\;{\mathrm{mm}}^{-1}$ [Fig. 7(a)]. For the blood vessel containing nNETs at depth of 500  μm, and blood vessels containing μNETs or nNETs at depth of 800  μm, the relationship between $\mu_{a,\mathrm{BV}}$ and % damage to blood vessels followed a sigmoidal behavior where below a certain threshold value of $\mu_{a,\mathrm{BV}}$ (and the corresponding $\mu_{a,\mathrm{NETs}}$) there was no damage to the vessel [Fig. 7(a)]. These results indicate that once $\mu_{a,\mathrm{BV}}$ exceeds $\approx 3\;{\mathrm{mm}}^{-1}$ with either μNETs or nNETs, % damage to blood vessels ranging in depth between 500 and 800  μm is maximized to the same level of ≈95%.

The therapeutic advantage of NETs in conjunction with 755-nm laser irradiation over current treatment at 585 nm without NETs was more evident with moderate skin pigmentation. For example, blood vessels with $\mu_{a,\mathrm{BV}}=6\;{\mathrm{mm}}^{-1}$, and containing μNETs or nNETs had >98% damage to vessels at either depths [Fig. 7(a)], whereas blood vessels without NETs irradiated at 585 nm had 34 and 11% damage to vessels at depths of 500 and 800  μm, respectively (Fig. 5).

**Fig. 8:**
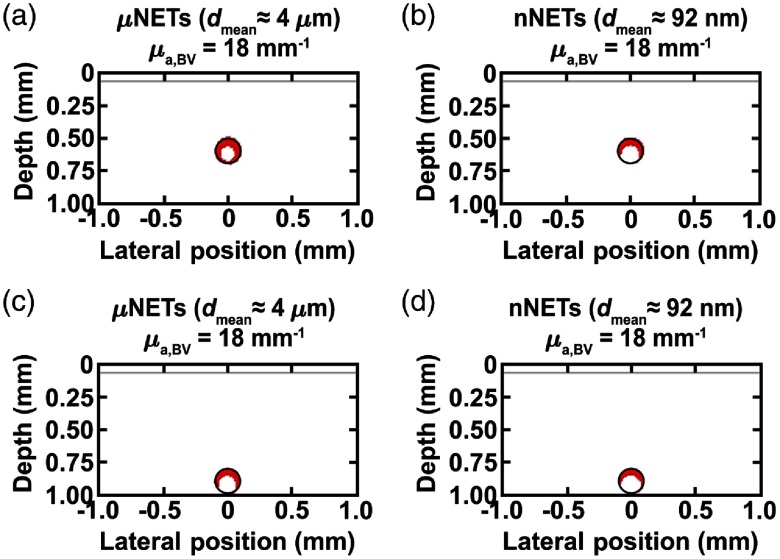
Damage profiles shown in red (Ω≥1) for blood vessels containing μNETs, or nNETs at $f_{\text{NETs}}=25\%$ with $\mu_{a,\mathrm{BV}}=18\;{\mathrm{mm}}^{-1}$, and located at depths of (a, b) 500 and (c, d) 800  μm below skin surface. Heavy skin pigmentation ($f_{\text{mel}}=50\%$), λ=755  nm, and $D_{\mathrm{th}}=3\;J/{\mathrm{cm}}^{2}$.

For heavily pigmented skin ($f_{\text{mel}}=50\%$), there was a dramatic decrease in damage to blood vessels containing NETs with $\mu_{a,\mathrm{BV}}=1$ to $6\;{\mathrm{mm}}^{-1}$ ($f_{\text{NETs}}=10\%$) in response to 755-nm laser irradiation at $D_{\mathrm{th}}=3\;J/{\mathrm{cm}}^{2}$ (results not shown). When $f_{\text{NETs}}$ was increased to 25%, blood vessels located at depths of 500 and 800 μm showed 60 and 36% damage, respectively, in presence of μNETs ($\mu_{a,\mathrm{BV}}=18\;{\mathrm{mm}}^{-1}$) [Figs. 8(a) and 8(c)]. At this value of $f_{\text{NETs}}$, blood vessels containing nNETs ($\mu_{a,\mathrm{BV}}=18\;{\mathrm{mm}}^{-1}$) showed 36 and 27% damage to vessels at depths of 500 and 800  μm, respectively [Figs. 8(b) and 8(d)]. For $\mu_{a,\mathrm{BV}}=18\;{\mathrm{mm}}^{-1}$, the corresponding ICG concentrations in the fabrication is ≈1258  μM [Eq. (12)].

These results demonstrate that blood vessels containing μNETs had greater therapeutic efficacy as compared to nNETs. The increase in % damage to a blood vessel containing μNETs is due to higher energy deposition values deeper into the blood vessels resulting from greater amount of light scattered in the forward direction by μNET (g=0.99, Table 2) as compared to nNETs (g=0.55, Table 2). Given their larger diameter, the optical behavior of μNETs is consistent with Mie scattering,^29^ allowing for higher forward scattering.

### 3.2 PWS Geometry Based on Patient OCT Image

**Fig. 9:**
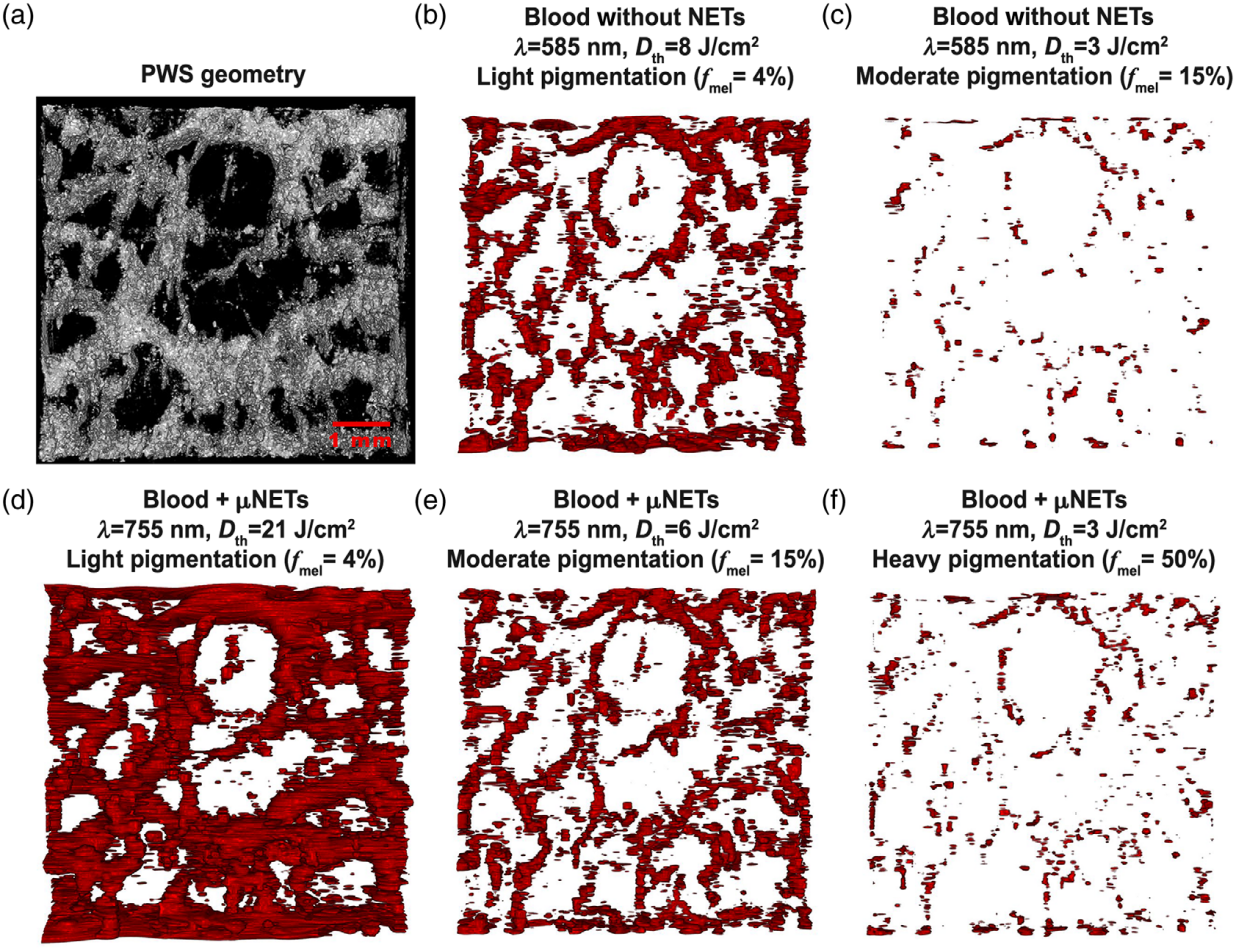
3-D rendering of (a) human PWS obtained by OCT (Video 1, MPEG, 2.3 MB [URL: https://doi.org/10.1117/1.JBO.23.12.121616.1]) and (b–f) damage profiles to PWS blood vessels. Red regions correspond to values of Ω≥1. (b, c) Damage profiles in response to 585-nm laser irradiation without NETs for light ($f_{\text{mel}}=4\%$) (Video 2) and moderate ($f_{\text{mel}}=15\%$) (Video 3) pigmentation levels, respectively (Video 2, MPEG, 2.3 MB [URL: https://doi.org/10.1117/1.JBO.23.12.121616.2]); Video 3, MPEG, 2.3 MB [URL: https://doi.org/10.1117/1.JBO.23.12.121616.3]. (d–f) Damage profiles in response to 755-nm laser irradiation in presence of μNETs for light ($f_{\text{mel}}=4\%$) (Video 4), moderate ($f_{\text{mel}}=15\%$) (Video 5), and heavy ($f_{\text{mel}}=50\%$) (Video 6) pigmentation levels, respectively (Video 4, MPEG, 3.3 MB [URL: https://doi.org/10.1117/1.JBO.23.12.121616.4]); Video 5, MPEG, 3.3MB [URL: https://doi.org/10.1117/1.JBO.23.12.121616.5]; Video 6, MPEG, 3.3 MB [URL: https://doi.org/10.1117/1.JBO.23.12.121616.6]. Parameters for patient PWS blood vessels containing μNETs were $\mu_{a,\mathrm{BV}}=6\;{\mathrm{mm}}^{-1}$, $f_{\text{NETs}}=10\%$ for (d), and $\mu_{a,\mathrm{BV}}=18\;{\mathrm{mm}}^{-1}$, $f_{\text{NETs}}=25\%$ for (e, f).

A 3-D rendering of the patient PWS geometry is shown in Fig. 9(a) (see also multimedia files). The PWS vessel diameters ranged from 60 to 650  μm with most around 200  μm and as deep as 1 mm below the skin surface. To test different skin pigmentations, epidermal layers were added to the geometry with light ($f_{\text{mel}}=4\%$), moderate ($f_{\text{mel}}=15\%$), and heavy ($f_{\text{mel}}=50\%$) pigmentations. Due to the greater therapeutic efficacy of μNETs for simulated PWS vessel geometries, we chose to use μNETs for patient PWS vessels with $\mu_{a,\mathrm{BV}}=6\;{\mathrm{mm}}^{-1}$ and $f_{\text{NETs}}=10\%$ for lightly pigmented skin ($f_{\text{mel}}=4\%$), and $\mu_{a,\mathrm{BV}}=18\;{\mathrm{mm}}^{-1}$ and $f_{\text{NETs}}=25\%$ for moderately ($f_{\text{mel}}=15\%$) and heavily ($f_{\text{mel}}=50\%$) pigmented skins. Damage profiles are shown for lightly, moderately, and heavily pigmented skin irradiated with 755 nm for PWS vessels with μNETs, or 585 nm for PWS vessels without NETs [Figs. 9(b)–9(f)].

**Fig. 10:**
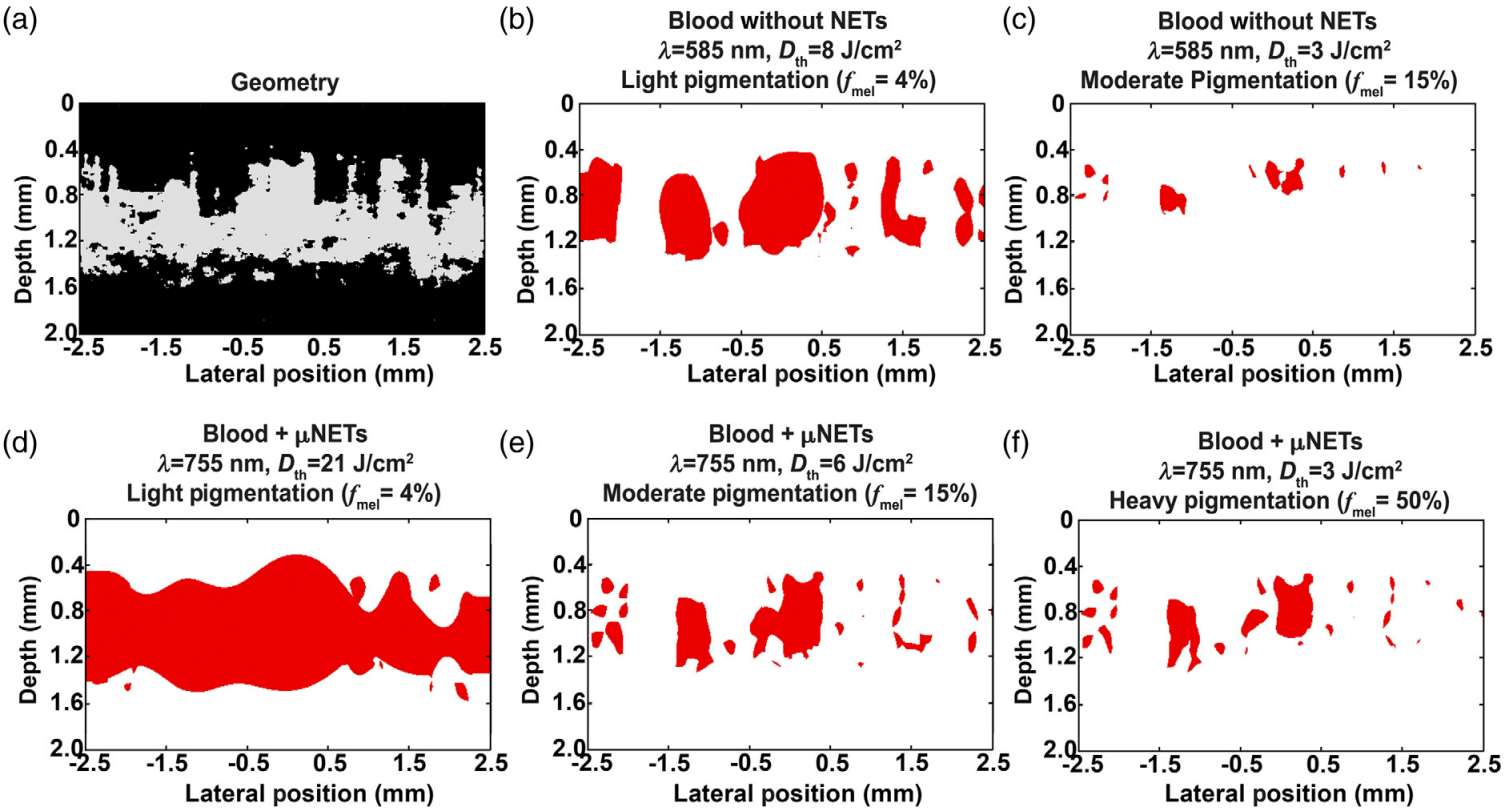
2-D x-z cross section of (a) human PWS obtained by OCT and (b–f) damage to PWS blood vessels. Red regions correspond to values of Ω≥1. (b, c) Damage profiles in response to 585-nm laser irradiation without NETs for light ($f_{\text{mel}}=4\%$), and moderate ($f_{\text{mel}}=15\%$) pigmentation levels, respectively. (d–f) Damage profiles in response to 755-nm laser irradiation in presence of μNETs for light ($f_{\text{mel}}=4\%$), moderate ($f_{\text{mel}}=15\%$), and heavy ($f_{\text{mel}}=50\%$) pigmentation levels, respectively. Parameters for patient PWS blood vessels containing μNETs were $\mu_{a,\mathrm{BV}}=6\;{\mathrm{mm}}^{-1}$, $f_{\text{NETs}}=10\%$ for (d), and $\mu_{a,\mathrm{BV}}=18\;{\mathrm{mm}}^{-1}$, $f_{\text{NETs}}=25\%$ for (e, f).

The therapeutic effectiveness of NETs in conjunction with 755-nm irradiation over 585-nm irradiation without NETs can be seen from these results. We calculated the % damage for vessels up to the depth of 1000  μm below the surface on a pixel-by-pixel basis within the simulated 3-D PWS geometry obtained by OCT imaging. This was accomplished by comparing each pixel in the 3-D damage profile with that of the 3-D geometry. The number of pixels showing values of Ω≥1 were summed and divided by the total number of pixels in the geometry to obtain an estimate of % damage. Lightly, moderately, and heavily pigmented skins showed % damage of 88%, 30%, and 4%, respectively, in presence of μNETs. For PWS vessels without NETs, there was 32 and 2% damage for lightly and moderately pigmented skins, respectively. In response to the irradiation parameters investigated, current therapeutic approach, based on 585-nm laser irradiation, is most effective in treating patients with light pigmentation by damaging blood vessels to a depth of ≈1.2  mm but still with some blood vessels remaining intact in the lateral direction [Fig. 10(b)]. Depth of damage is reduced to ≈0.8  mm and accompanied by a reduction in lateral vascular damage in moderately pigmented PWS skin [Fig. 10(c)].

Under the parameters investigated, laser irradiation at 755 nm in conjunction with μNETs can increase the damage depth to ≈1.4  mm and also induce complete damage to the vascular plexus in the lateral direction in patients with light pigmentation [Fig. 10(d)]. For patients with moderate and heavy pigmentation levels, blood vessels to depth of ≈1.2  mm can be damaged when using 755-nm laser irradiation in conjunction with NET, but the extent of lateral vascular damages decreases [Figs. 10(e) and 10(f)]. These results suggest that similar to the optical response of single blood vessel, there may be shadowing effects to reduce the energy deposited to deeper blood vessels.^49^ Such effects can occur if vessels are as close as a few microns.^50^ Optimizing the ICG concentration in NETs may provide a means to allow for more uniform light energy distributions.

Use of free ICG in conjunction with pulsed 808- and 810-nm laser irradiation for treatment of patients with PWS has been reported.^11^^,^^12^ While promising clinical results have been reported in these studies, delivering NETs to PWS blood vessels is more advantageous than free ICG as it provides a method to increase the circulation time of ICG, and can consequently, elongate the therapeutic window of time over which laser irradiation can be performed. For example, nanoconstructs (≈80  nm diameter) composed of poly (lactic-co-glycolic acid) core coated with erythrocyte-derived membranes were retained in blood for 3 days with circulation half-life of ≈8  h in mice.^51^ Piao et al.^52^ reported that the circulation half-life of gold materials cloaked with erythrocyte membranes (≈90  nm diameter) was ≈9.5  h. Increased circulation time of erythrocyte-coated particles can be attributed to the presence of “self-marker” membrane proteins to allow evasion by immune cells.^53^^,^^54^ One important “self-marker” membrane protein on the surface of the erythrocytes is CD-47 glycoprotein, whose interaction with immunoreceptor signal regulatory protein alpha (SIRPα), expressed by macrophages, results in inhibition of phagocytosis.^55^ We previously demonstrated that CD-47 remains on the surface of the NETs,^56^ providing a mechanism for increased circulation time of NETs. Furthermore, NETs can serve as a biocompatible and nontoxic platform for delivery of ICG. In a recent study, Rao et al.^57^ reported the absence of systemic toxicity at 15 days postintravenous injection of erythrocyte membrane-coated upconversion nanoparticles in mice.

Results of this theoretical study indicate that use of NETs in conjunction with pulsed 755-nm laser irradiation can provide a personalized approach for effective treatment of PWS blood vessels by customizing the optical properties of NETs to match the type of vascular plexus (e.g., depth, size, and distribution of blood vessels) and induce the necessary photothermal effects. The theoretical model presented in this study can be used in guiding the fabrication of NETs with an appropriate level of ICG and development of laser dosimetry parameters.

## 4 Conclusions

Using theoretical models, we have demonstrated that NETs can be beneficial for NIR laser treatment of PWS. NETs can be fabricated with patient-specific optical properties to allow for a personalized treatment based on the size and depth of the blood vessels as well as the pigmentation of the individual’s skin. The use of NIR lasers in combination with NETs addresses key challenges in vascular phototherapy by reducing epidermal damage and increasing penetration to deeper blood vessels.
